# Stress Classification Using Brain Signals Based on LSTM Network

**DOI:** 10.1155/2022/7607592

**Published:** 2022-04-28

**Authors:** Nishtha Phutela, Devanjali Relan, Goldie Gabrani, Ponnurangam Kumaraguru, Mesay Samuel

**Affiliations:** ^1^Department of Computer Science and Engineering, BML Munjal University, Gurugram, India; ^2^College of Engineering, Vivekananda Institute of Professional Studies Technical Campus, New Delhi, India; ^3^Department of Computer Science, International Institute of Information Technology, Hyderabad, India; ^4^Computing and Software Engineering, Arba Minch University, Arba Minch, Ethiopia

## Abstract

The early diagnosis of stress symptoms is essential for preventing various mental disorder such as depression. Electroencephalography (EEG) signals are frequently employed in stress detection research and are both inexpensive and noninvasive modality. This paper proposes a stress classification system by utilizing an EEG signal. EEG signals from thirty-five volunteers were analysed which were acquired using four EEG sensors using a commercially available 4-electrode Muse EEG headband. Four movie clips were chosen as stress elicitation material. Two clips were selected to induce stress as it contains emotionally inductive scenes. The other two clips were chosen that do not induce stress as it has many comedy scenes. The recorded signals were then used to build the stress classification model. We compared the Multilayer Perceptron (MLP) and Long Short-Term Memory (LSTM) for classifying stress and nonstress group. The maximum classification accuracy of 93.17% was achieved using two-layer LSTM architecture.

## 1. Introduction

Stress can be triggered by the change in the body's emotional response to various situations such as depression, anxiety, anger, grief, guilt, low self-esteem, etc. It can be classified as positive stress (eustress) or negative stress (distress) [1]. Stress is the root cause for a variety of mental health problems like depression and dementia and has an adverse effect on a person's performance [2]. Issues related to stress are rising exponentially worldwide; therefore, the detection and quantification of stress are of utmost importance [3].

There are different ways to measure stress levels. Traditionally, the stress level of an individual has been calculated only through self-reports [4]. Some standard questionnaires are available, and the answers filled by subjects to those questionnaires are mapped to some predefined scales. Each question is assigned some score based on the answer given, and the total score is calculated from all the questions answered [5]. Different standard scales are used in clinical settings through which stress can be quantified [6–8] but they are subjective indicators. Moreover, in developing countries, people do not prefer to go to psychologists and mental health clinics, due to the social stigmas associated with it. Thus, there is a need for a system that can automatically classify the subjects into stress and nonstress. It may also help to develop preventive measures, for instance, to make the people aware about their mental health [9]. Stress measurement based on questionnaires, facial expressions (blink rate of the eye, voice etc.), social media posts, etc. are either subjective or challenging to validate [10–14].

It has also been observed that stress has a significant effect on specific physiological parameters like skin conductance, blood pressure, and brain signals [15, 16]. Various physiological signals acquired from different sources such as electroencephalography (EEG), Functional Magnetic Resonance Imaging (FMRI), and Positron Emission Tomography (PET) were used for the detection of stress [17–19]. Among these, EEG has gained acceptance in monitoring stress levels as it is noninvasive and nonexpensive and gives very high temporal resolution [20]. There are certain attributes (physical, physiological, etc.) that can help in classifying stress from nonstress individuals. But there is no direct metric for stress measurement.

Various attempts are made where the classification was performed using different features. But different features sets yield different results. Recently deep learning has been widely used in the domain of stress recognition through EEG [21, 22] as it can directly take input from raw data and identify the most prominent features automatically without any feature engineering and preprocessing [23, 24]. But according to [25], although deep neural networks are capable of learning features, in order to yield high performance it is better to do feature extraction beforehand. Moreover, deep learning model is data hungry. Thus, to build a reliable stress detection system with sophisticated feature extraction tool is highly prized. At the same time it is not straightforward to know the optimal features which can classify the stress level with high accuracy. Moreover, the type and number of features to be extracted highly depend on the type of headband. EEG has advantages of low cost, high temporal resolution, and ease of use. It is one of the most used techniques for stress and other mental states' assessment [26, 27].

To this end, we propose a stress classification system that utilizes the direct FFT signals provided by Muse headband for stress identification. We used inexpensive Muse headband for the acquisition of EEG signal as numerous studies have reported its applicability in identifying the brain activities of an individual [28, 29]. We build a simple stress classification system which uses minimum number of sensors. In literature, various systems are proposed which uses different set of features for stress classification but in our present work we used direct signal provided by the device which reduces the computational cost for calculating the features manually. In one of the previous works, authors used LSTM recurrent neural network for emotion classification with 4-channel EEG device [30]. To the best of our knowledge, we are the first to explore Long Short-Term Memory (LSTM) for stress classification using just 4 EEG electrodes. Thus, the major contributions of this paper are summarized as follows:

- Creating a data set of recorded EEG signals which were acquired while participants watched the video clip (as a stress elicitation material) to infer the stressed state of the subject.
- Building a model and comparing Multilayer Perceptron (MLP) and LSTM based architecture for the classification of stress data.

The content in this paper is structured as follows: The literature review corresponding to this topic is given in Related Work section. The requisite background for understanding the paper is elaborated in Background section, the proposed methodology and experimental setup are described in Proposed Methodology section. Various metrics used to gauge the performance of the proposed model are mentioned in Performance Measures section. Results and Discussion section details out the results of the experiment conducted for the classification of stress. Conclusion and Future Work section contains some conclusive remarks and a few pointers on future work.

## 2. Related Work

Notably, a psychologist can provide a huge amount of knowledge in identifying if a person is stressed. But in the absence of a psychologist, identifying features that are representative of stress from the collected EEG data becomes a challenge. Most of the work on stress detection has relied on various hand-crafted features [31, 32], so there is a definite need to explore the area more. Thus, keeping in view the health problems that are associated with increasing stress levels, it becomes extremely important to identify that a person is stressed at an earlier stage.

Recently deep learning has been widely used in the domain of stress recognition through EEG [21, 22]. A detailed review of deep learning techniques for classification tasks using EEG signals is reported in [33]. The advantage of using deep learning is that it can directly take input from raw data and identify the most prominent features automatically without any feature engineering and preprocessing [23, 24]. As a result, the difficulty of selecting the best appropriate preprocessing algorithm and feature selection methods has been overcome, making this more applicable. But according to [25], although deep neural networks are capable of learning features, it is better to do feature extraction beforehand. This is because EEG signals contain noise and interference. Furthermore we need a lot of data for training purpose in building a deep learning model.

Stress can be detected in natural setting or controlled lab setting. The authors of [21] attempted to record the pattern of workers' brain waves at a construction site (natural setting) when they were under stress. Their aim was the early detection and mitigation of stress for the construction workers. To obtain the ground truth, the saliva of the workers was collected which contains a hormone called cortisol responsible to regulate stress. The authors conducted this study on 9 construction workers using 14 electrodes' mobile EEG device. The intrinsic signal artifacts were removed by using Independent Component Analysis (ICA) and the extrinsic signals artifacts were removed by using low pass filter, high pass filter, and notch filter with appropriate frequencies. The authors have proposed a system which utilizes 14 electrodes and built a model using convolutional deep learning network and a fully connected deep neural network architecture for binary stress classification of stress. The accuracy reported by using fully connected DNN was 86.62%. Their DNN architecture consisted of 2 hidden layers with 83 neurons in the first layer and 23 neurons in the second hidden layer, while their approach reported an average increase in the stress classification accuracy as compared to their previous approach of using SVM by [34]. A major limitation of this study was smaller number of participants so it becomes hard to generalize the results. Also collecting saliva might not be favourable to the subjects under study. They have also not specified if a medical practitioner was in their team to label the stress level based on collected saliva sample. Also, testing the internal validity in their experiments is a challenge because there is no mention of recording multiple sessions with same subject.

Under controlled lab setting, various stress elicitation material was used such as evoked emotional stress through multitasking [35], Paced Auditory Serial Addition Test (PASAT) [36], and Stroop Color Word Test (SCWT) [37].

A stressed emotion dataset called Multimodal Dataset of Stressed Emotion (MuSE) has been presented in [22] to study the correlation between occurrence of stress and the presence of affect. They have considered stress as one of the confounding factors in influencing the psychological state of a person. They have collected data from 28 students during the final exams and after the exams period to create datasets for stress and nonstress, respectively. The experiment is comprised of a series of the following events: emotional stimuli presentation, video watching, and emotionally evocative monologues. Perceived Stress Scale (PSS) was used to get self-reported scores of stress and Self-Assessment Manikin (Sam) was used for emotional assessment. They used a paired *t*-test to infer that the average PSS scores obtained from the two groups were significantly different. A considerably large recording of 45 minutes was used. The novelty in their experiment design was the use of different emotional elicitation materials across all sessions, even though the emotional dimension being captured was the same. They have used various unimodal deep neural networks and also a multimodal ensemble for the modelling valence and activation. They have segmented the video dataset with a window size of 1 second and an overlap of 0.5 seconds. Out of the diverse set of features that have been used in this work, the visual and physiological modalities perform the best for stress elicitation while being influenced emotionally. The reported accuracy from their work is 70%. Other datasets available for stress detection include the one in [38]; it is a real world biometric dataset collected from nurses working in a hospital at the time of COVID-19. The physiological variables measured in this dataset include EDA, heart rate, GSR, and accelerometer reading. WESAD is another publicly available dataset collected in a controlled lab setup. It contains physiological and motion related data of 15 participants [39]. Various machine learning algorithms have been used to differentiate stressed, neutral, and amused emotion. Authors in [40] have introduced another large scale dataset of stress using physiological signals. Reference [41] is a multidomain social media dataset for identifying stressed state of an individual.

The use of stress elicitation material in the proposed work is inspired by work by authors in [31], who use EEG, arousal, and valence dimensions to measure stress during video watching. Videos contain audio as well as visual stimuli that have more effect on the brain as compared to using a single stimulus.

The application of LSTM network for classification of brain signals has been reported by [42–44].LSTM with attention mechanism has been used by [45] to develop cross-subject generalized solution for classifying limb (hand) movements using EEG. They have used frequency as well as time based features as input to LSTM network and obtained an accuracy of 83.2%. In [46] the authors reported the highest accuracy of 92.8% in context of driver stress classification tasks (at different weather conditions and other ambient factors) using single physiological signal, i.e., electrocardiogram (ECG) signal. They used LSTM and CNN to detect driver's stress.

## 3. Background

This section presents the relevant background details required for understanding of the proposed stress classification model.

### 3.1. MLP

Multilayer Perceptrons are the type of feedforward neural networks that are widely used because they operate fast, can efficiently work on small training data sets, and are easy to implement. A typical MLP architecture consists of an input layer, series of hidden layers, and an output layer. The input layer consists of neurons equivalent to the number of features in the input data. The hidden layer processes the information selectively from input layer. It accomplishes this by associating weights and biases with the input features. There is no fixed rule to obtain the number of neurons in the hidden layer. It is a hyperparameter and has to be tuned with multiple trials. The output layer consists of the number of neurons depending on the classification task. For instance, if the problem consists of a binary classification task, then the output layer will contain only one neuron.

### 3.2. LSTM

**Figure 1:**
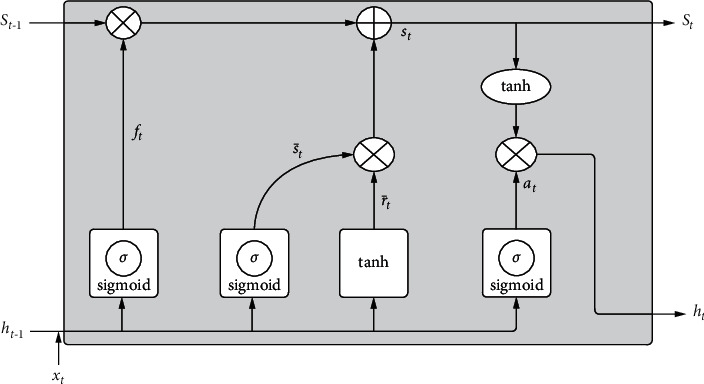
The basic architecture of an LSTM cell containing a forget gate, an input gate, and an output gate.

LSTMs were introduced by Hochreiter and Schmidhuber in 1997. The special feature about this kind of neural networks that differentiates them from Recurrent Neural Networks (RNNs) is that they can learn long term dependencies. It is one of the best algorithms to work with sequence data along with an additional feature of having a memory element [47]. This memory element enables LSTM to remember the previous sequence of steps. It overcomes the difficulty of vanishing gradient, faced with RNN by a slight modification in the structure.  Figure 1 shows the elementary architecture of a cell in LSTM [48]. The enabler of this cell is the parallel line shown in the upper part of Figure 1(*C*_*t*_).

LSTM can let selected information to flow through it with the help of this cell state. This feature comes with the help of three logic gates. Each of these gates gets input from the sigmoid activation function. The forget gate (*f*_*t*_) is the first gate that selects the information that needs to be discarded from the cell. The equation for the forget gate is

(1) $$\begin{array}{c} f_{t}=s\left(Wt_{f}\left[h_{t-1},x_{t}\right]+bs_{f}\right). \end{array}$$

The input gate is the second gate; its functionality can be explained in two parts (*i*_*t*_) and $\left({\tilde{c}}_{t}\right)$: The first part is explained through (2) which involves a *sigmoid* function that computes any update in the previous values. The second part involves the tanh function as shown in (3) which creates a vector of new updated values.

(2) $$\begin{array}{c} i_{t}=s\left(Wt_{i}\left[h_{t-1},x_{t}\right]+bs_{i}\right), \end{array}$$

(3) $$\begin{array}{c} {\tilde{c}}_{t}=th\left(Wt_{c}\left[h_{t-1},xt\right]+bs_{c}\right). \end{array}$$

Then the state of the old cell *C*_*t*−1_ is replaced by the new cell state by removing the information generated by the forget gate in (1). *C*_*t*_ in (4) denotes the updated cell state.

(4) $$\begin{array}{c} C_{t}=f_{t}*C_{t-1}+i_{t}*{\tilde{C}}_{t}. \end{array}$$

Finally, the output is surpassed from a sigmoid layer and then a tanh layer to classify.

(5) $$\begin{array}{c} o_{t}=s\left(Wt_{o}\left[h_{t-1},x_{t}\right]+bs_{o}\right),\\ h_{t}=o_{t}*th\left(C_{t}\right). \end{array}$$

After the above steps, the cell state is updated. Lastly, output of current state is computed by taking the values of updated state of the cell state and also values from the sigmoid layer that determines the components of the cell state that need to be included in the output. The terminologies used in the above equations are described as below:

1. The activation function *s* is sigmoid that suppresses the values in the interval (0, 1).
2. The activation function *th* is hyperbolic tangent that suppresses the values in the interval (−1, 1).
3. The weight matrices are represented by *Wt*_*f*_, *Wt*_*i*_, *Wt*_*c*_, *Wt*_*o*_.
4. The input values are contained in a vector called *x*_*t*_.
5. The bias vectors are denoted by *bs*_*f*_, *bs*_*i*_, *bs*_*c*_, *bs*_*o*_.

It may be noted that the last sigmoid layer will classify the data into stressed or nonstressed group.

## 4. Proposed Methodology

### 4.1. Device Description

**Figure 2:**
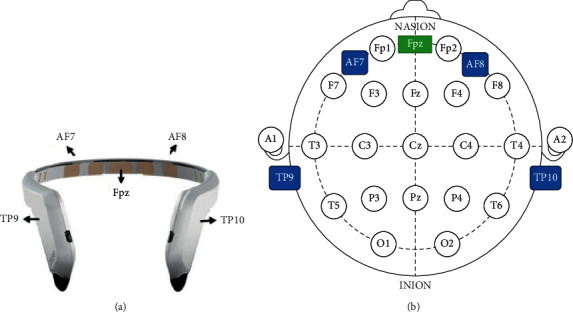
(a) Muse headband for measuring the activity of the brain via four electrodes: AF7, AF9, TP9, and TP10. (b) 10–20 system of electrode placement (source: [32]).

We used Interaxon Muse brain sensing headband with 4-channel EEG devices to acquire brain signals. It is a low cost device as compared to the medical EEG devices used by doctors. This headband is easy to adjust, does not consist of any wires, and does not need medical supervision. The Muse headband consists of four dry electrode channels (TP9, AF7, AF8, and TP10) working at global standard of 10–20 coordinates. AF7 and AF8 are the two forehead electrodes while TP9 and TP10 are two ear electrodes (see Figure 2). The device outputs the brain waves into various frequency ranges, namely, (i) delta, (ii) theta, (iii) alpha, (iv) beta, and (v) gamma.

Delta (0–4 Hz) is the default brain wave signal. These signals are observed when we sleep, in clam state, and when a person is in comma. These are affected in case of serious brain injury. Theta (4–8 Hz) is a transitory brain wave between the slower delta and the comparatively faster intermediate brain waves, i.e., beta and alpha. It is said to be associated with creativity. Beta/Theta ratio is useful to determine the activeness of a person. Theta reflects activity from the limbic and hippocampal regions. Alpha (8–12 Hz) is associated with meditation. Alpha has been associated with wakeful mindfulness. They are strongest over occipital (back of the head cortex) and frontal cortex. Beta waves (>12 Hz, 13–21 Hz), on ther hand, are associated with high analytical thinking. A lot of beta activity happens in the frontal lobe region (AF7 and AF8). Frontal lobe is the area connected with executive function which is in turn responsible for figuring things out. Beta is highly associated with high performance and anxiety. Gamma waves are very fast oscillations (>30 Hz, 31–80 Hz) and are associated with higher level information processing like integrating thoughts.

### 4.2. Data Collection

For this experiment, signals from 40 subjects were acquired and out of these the data from 5 subjects were corrupted because the connection between electrodes and scalp was loose. This was identified during the manual inspection as the signal has *NAN* value for these subjects. Finally, 35 subjects were selected (18 males and 17 females) from the age group between 23 and 55 years. All the subjects were healthy and did not have any kind of neurological disorder. For each subject EEG signals were recorded. The subjects of our experiment were instructed not to consume any caffeine product at least 12 hours prior to the start of the experimental process because caffeine is found to interfere with brain activity [49].

The data used in the analysis has been collected from participants wearing the Muse headband as shown in Figure 2. The headband was adjusted to the comfort of the participant. Subsequently, they were shown four movie clips from the list shown in Table 1. The use of stress elicitation material in proposed work is inspired by work by authors in [31], who use EEG, arousal, and valence dimensions to measure stress during video watching. These authors have used the circumplex model of affect. This model maps stress to high arousal and low valence. Stress causes high arousal because the mind is activated, but the activation is not pleasant, so the valence is low. Table 1 contains the information about the clips shown. These clips were specifically chosen from Indian film clips because the subjects were able to relate better to these clips. A buffer of 2 minutes was incorporated after each clip. This was done to relax the participant from the effect of the stressed and nonstressed videos. The venue for the experiment was an isolated room. The participants were asked to switch off all electronic devices present with them so that there is minimum interference from these devices on the EEG signals [50].

While watching the video clip, the participant's EEG signals were recorded. After watching each movie clip, the participant filled an assessment form to infer the level of stress induced by each of these clips. The subjects were asked to complete the State Anxiety questionnaire [8]. State Anxiety is a multiscale questionnaire which we have used to test if the subject has experienced stress after watching the video clip. It has total 20 items. Each of these items is used to infer the feeling of the subject at the current moment. The responses of these questions were taken on a 4-point Likert Scale (1- Not at all stressed, 2-Some what stressed, 3-Moderately stressed, 4- Very much stressed). The answers from all the respondents were evaluated according to the standard scoring key of State Anxiety scale. The scores of this questionnaire were generally higher after watching the stress inducing videos and lower after watching the nonstressed videos. This data was used as a ground truth to label each instance of EEG recording from the respective person. The procedure followed was in accordance with Helsinki declaration. Also, the participants were informed about the procedure in advance and a consent form was signed by them before starting the experiment.

It may not be necessary that every participant will get stressed after watching the stress video enlisted by the authors; it depends on an individual's stress coping ability [51].

The score of State Anxiety form varies from 20 to 80. We calculated the average scores of participants from the questionnaire, similar to work performed in [32]. The participants whose score was greater than the average score were categorized in the stress group and the others in nonstressed group. Thus, the authors explicitly used average score in the questionnaire as the threshold. Higher scores correlate with greater anxiety.

The records in the data set were labelled as stressed if the score obtained in the State Anxiety scale is greater than or equal to 50 and nonstressed if the score is less than 50. Authors in [52] showed that stress lies in the top left quadrant of the circumplex model of affect. This quadrant is characterized by high arousal and low valence. The meaning of arousal and valence was explained to the subjects and they were asked to rate the video in terms of arousal and valence. The range of values of arousal and valence for identifying the stressed and nonstressed states were similar to those used by [31]. The data of the participants which did not represent stressed and nonstressed behaviours (in terms of arousal, valence, and State Anxiety) corresponding to the stressed and nonstressed stimuli was discarded. This was done to ensure that the ground truth of the stress classification model is correct. There were 2 such subjects so their data was discarded.

### 4.3. Experimental Setup

The LSTM architectures were built-in Keras 2.0.9 using TensorFlow backend in Python 3.6. Using LSTM with Keras requires the input in three dimensions (samples, time steps, and features). Our long univariate time series data sequence was reshaped into smaller segments and then fed into Keras. These segments/subsequences can be overlapping or nonoverlapping. In our work, we split our long time series data into overlapping subsequences to increase the number of training samples. This also helps to capture the dependence between individual subsamples of data. A smaller window size leads to the model getting trained faster and makes the model more robust and able to capture more information from individual slices of a single sequence of data. As EEG is a fast and dynamic signal which changes within a short duration of time, it is important to process the data in small chunks [53]. Thus we took a small window size.

We used the LSTM network for the binary classification of stress due to its associated advantages. LSTM is used for sequence classification problems and has ability to extract significant temporal information from physiological signals [30, 54]. Moreover, the LSTM network makes predictions based on the individual time steps of the input sequence data.

The dataset was divided into similar length sequences, which is an important step while the training process as input sequences should be of the same length. Thus, the length of the EEG recording for each of the trials was 80 seconds. Each second of the recording has 50 data points. So a total of 4000 data points exist for each of the trials. A window size of 20 was selected with an overlap of 50% to break down this sequence into smaller segments.

A minibatch gradient descent algorithm was trained on these smaller segments with a batch size of 32. Minibatch gradient descent is the most common implementation of gradient descent. The frequency of update process after each iteration is faster as compared to batch gradient descent. This helps to avoid local minima by giving a robust convergence. Minibatch sizes, commonly called “batch sizes” for brevity, are often tuned to an aspect of the computational architecture on which the implementation is being executed, such as a power of two that fits the memory requirements of the GPU or CPU hardware like 32, 64, 128, 256, and so on. The smaller values of this minibatch size give a learning process that converges quickly at the cost of noise in the training process. Large values give a learning process that converges slowly with accurate estimates of the error gradient. A good default value for batch size is 32 [55]. Reference [56] has also concluded that information on emotions contained in the EEG signal may be better described in shorter time segments.

We started with one LSTM layer (LSTM 1) containing 8 neurons and gradually included a second LSTM layer (LSTM 2) containing 16 neurons and subsequently another LSTM layer (LSTM 3) containing 24 neurons. There was not much difference in the accuracy obtained between the LSTM 2 and LSTM 3 model. Thus we concluded LSTM 2 model was sufficient to classify EEG signals as the accuracy does not improve further, which is in accordance with the observation made in [25].

**Figure 3:**
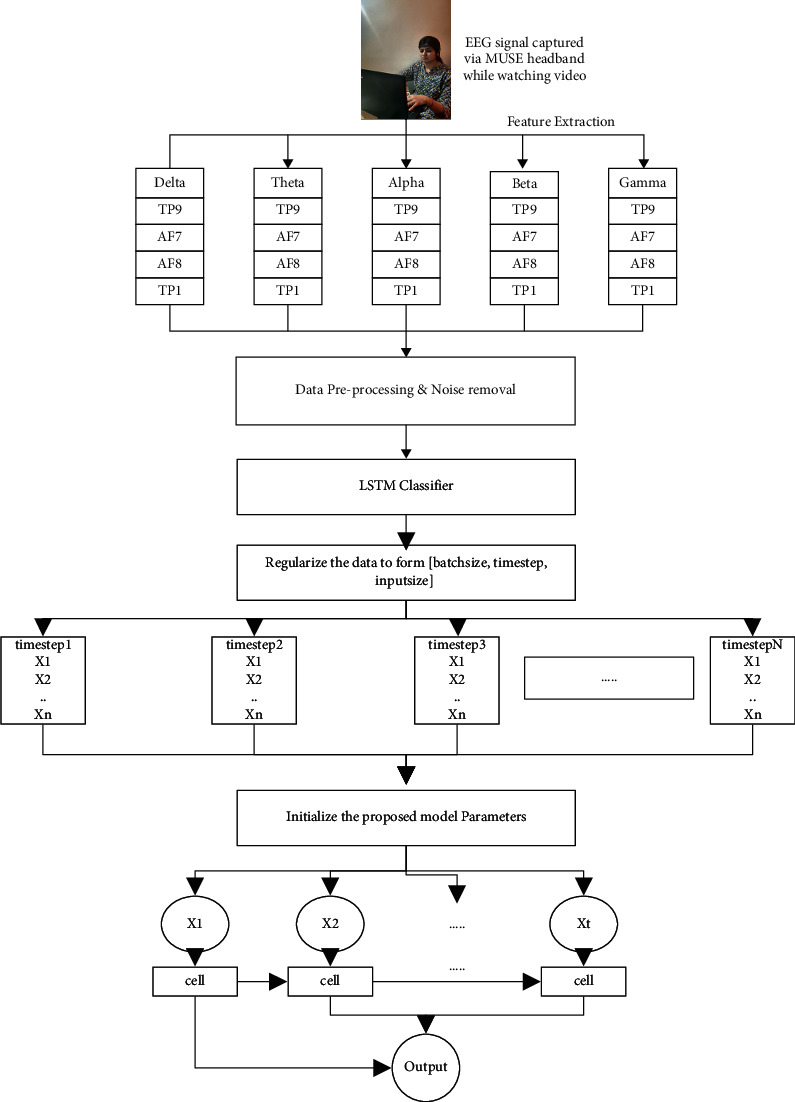
Proposed model for stress classification using EEG signals.

Our model also contained a fully connected dense layer for classification [57]. Because deep learning networks tend to overfit, we also use a dropout layer to avoid the model learning noise [58, 59]. We used sigmoid activation function and one neuron in the output layer since we were doing binary classification (stressed and nonstressed). Since our training data contains 20 features/signals (4 signals (corresponding to signal from 4 regions: TP9, AF7, AF8, and TP10) for each of the 5 brain frequency bands) as shown in Figure 3, 20 neurons were used in the input layer.

Also, the state-of-the-art *Adam* optimizer was used to regulate the change in step size during the learning process. To evaluate how well the trained model has performed, binary cross entropy loss function was used because we had only one neuron in the output layer. The values of parameters obtained after hyperparameter tuning have been listed in Table 2. The values of these hyperparameters have been chosen based on cross validation. The parameters which performed best have been chosen.

## 5. Performance Measures

The following performance measures have been identified to evaluate the performance of the stress classification framework. The count of true positive cases is indicated by TP. The count of true negative cases is represented by TN. False positive cases are shown with FP and false negatives with FN.

### 5.1. Confusion Matrix

This metric gives information about actual labels for the data and the labels that are obtained through the classification model used. The diagonal elements of this matrix give the correctly classified records and the off-diagonal elements give the misclassified records.

### 5.2. Accuracy

The accuracy of the classifier is calculated based on

(6) $$\begin{array}{c} \text{Accuracy}=\frac{\text{TP}+\text{TN}}{\text{TP}+\text{TN}+\text{FP}+\text{FN}}\times 100. \end{array}$$

This metric gives an estimate of the fitness of the classifier used.

### 5.3. Specificity

This metric maps the actual nonstressed instances to those identified by the classifier. This in turn is calculated using

(7) $$\begin{array}{c} \text{Specificity}=\frac{\text{TN}}{\text{TN}+\text{FP}}. \end{array}$$

### 5.4. Precision

The motive of calculating precision is to find the total number of correct positive predictions from the total number of positive predictions using

(8) $$\begin{array}{c} \text{Precision}=\frac{\text{TP}}{\text{TP}+\text{FP}}. \end{array}$$

### 5.5. Recall

This metric corresponds to the number of samples correctly classified as being stressed. Mathematically, it is calculated using

(9) $$\begin{array}{c} \text{Recall}=\frac{\text{TP}}{\text{TP}+\text{FN}}. \end{array}$$

### 5.6. F1-Score

This particular metric uses the harmonic mean of precision and sensitivity and is preferable when the dataset is unbalanced as the minority class also carries significant amount of information. It is calculated using

(10) $$\begin{array}{c} F1-\text{score}=2*\frac{\text{Precision}*\text{Recall}}{\text{Precision}+\text{Recall}}. \end{array}$$

### 5.7. Mann-Whitney Test

Reference [60] is a statistical test used to check the significance of two analytical results by comparing their median values. If *p*-value is less than 0.001, it can be deduced that the classifiers used are highly significant. Otherwise the result is insignificant.

The proposed model is depicted in Figure 3. After collecting data, it is preprocessed into a form compatible with LSTM architecture. The detailed working of LSTM has already been described in Section 3.

## 6. Results and Discussion

The classification of the mental stress of the participants into stressed and nonstressed categories was achieved with the help of MLP and LSTM. State-of-the-art parameter values (Table 2) were used for the implementation of MLP and LSTM. These algorithms were run on the same system to reduce experimental error.

The performance of the stress classification model is measured with the help of the following training-testing data partitions: 50–50, 60–40, 70–30, and 10-fold cross validation. The data was split into various proportions for testing and training in all these techniques. 10-fold cross validation was used to introduce randomisation into the training and test set choice. For each of the validation techniques used, Table 3 shows a confusion matrix consisting of two rows and two columns corresponding to each of the classifiers used.

The diagonal elements of this matrix indicate the instances in the dataset that were correctly classified. The nondiagonal elements constitute the wrongly classified instances. The high values of the diagonal elements indicate that our model is correctly able to differentiate stressed and nonstressed classes. As depicted in Table 4, LSTM 2 gives the average accuracy of 91.96% and maximum accuracy of 93.17% using 10-fold cross validation, which is much higher than the accuracy obtained through MLP. This result demonstrates the capabilities of LSTM to remember long term information from the sequential data.

**Figure 4:**
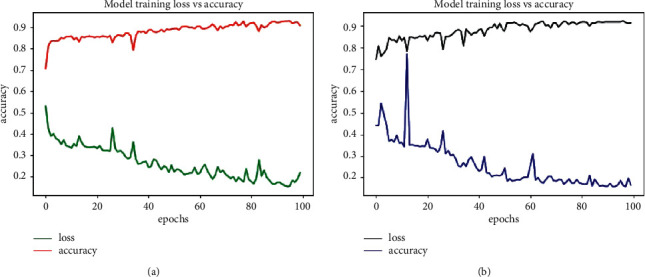
Model loss and accuracy with LSTM 2 architecture using 10-fold cross validation. (a) Train data. (b) Test data.

Figure 4 denote the train and test accuracy and loss obtained by deploying LSTM 2 model on our data. Also, Table 5 illustrates the other performance metrics used which show the robustness of the proposed approach. Higher recall and precision of our model showed that it gives less false negatives and false positive, respectively. High specificity denotes the true negative rate; i.e., a person classified as nonstressed was actually nonstressed.  Table 6 shows the values of Mann-Whitney test computed on the results given by LSTM 2. These denote that the improved accuracy results obtained with LSTM are highly significant.

Table 7 shows the comparison of our proposed system with other state-of-the-art approaches available in the literature. It is evident from the comparison that our approach, which utilizes LSTM based model, is better in terms of classification accuracy. The work proposed by authors in [63] got a better stress classification accuracy using deep learning methods, but the number of subjects in their study was less than one-third of the number of subjects used in our proposed study. Also they have used saliva to elicit ground truth which is not easy because the saliva sample has to be sent to laboratory to extract the level of cortisol (stress hormone) present. It is the level of cortisol which further gives information about the stressed state of the person. The highest accuracy achieved by the proposed method is 87.22%, 89.28%, 90.33%, and 93.17% for 50–50, 60–40, 70–30, and 10-fold cross validation, respectively. Also, the average accuracy achieved is 85.79%, 82.75%, 83.14%, and 86.55% for the abovementioned training-testing data partitions, respectively, as shown in Table 4. These results indicate that LSTM is a good candidate for classifying stress-related brain signals data.

## 7. Conclusion and Future Work

This study proposes a system for stress classification using EEG signal acquired from Interaxon Muse 4-channel commercially available headband device. The EEG signal was recorded from stress and nonstress subjects. The stress elicitation had been done using Hindi movie clips. Headband device provides the FFT signal which was directly used for classification task. We compared the MLP and LSTM model for classifying stress from nonstress. 35 participants took part in this experiment and watched film clips targeted to elicit the emotion of stress. The classification of stress was achieved with a maximum accuracy of 93.17% using LSTM 2 (with 2 LSTM layers). These results demonstrated an improved performance over state-of-the-art methods utilizing EEG signals. Moreover, it is important to mention here that direct comparison is not possible with the state-of-the-art methods due to difference in experimental setup, number of subjects, difference in the subjects, etc.

The proposed stress classification method, from the best of our knowledge, is the first to observe the effect of video watching on mental stress using a commercially available EEG headband with 4 electrodes. This research work is a proof of concept to illustrate the applicability of an EEG headband to reliably identify the stressed state of a person. In future the authors will extend the work by conducting the experiment with more number of subjects.

## Figures and Tables

**Table 1:** Summary of the excerpts from films shown for stress classification.

Category	Name of the film	Duration (sec)	Clip content

Nonstressed	3 Idiots	80	The kick of a stillborn child creates amusement among the surrounding people
	Taare Zameen Par	124	A music teacher delights his students with a motivational song
Stressed	Rang de Basanti	117	The nation mourns during the cremation of a warrior
	Kal Ho Na Ho	96	Friends converse with another friend who is about to die

**Table 2:** Parameters for LSTM models chosen after hyperparameter tuning.

Parameter	Values

Number of input features	20
Number of output features	1
Number of LSTM layers	2
Number of hidden units in LSTM layers	8 and 16 (only for two-layer LSTM)
Activation function	Sigmoid
Optimizer	Adam
Loss function	Binary cross entropy
Batch size	32
Window size	20
Epochs	100
Dropout value	0.2

**Table 3:** Confusion matrix for stress classification: Diagonal elements contain the TP and TN values, respectively, and the nondiagonal elements contain the FP and FN, respectively.

Validation techniques	Classifier					
	MLP		LSTM 1		LSTM 2	

50–50	4300	990	4700	600	5200	360
	1700	2010	2108	1300	790	2650
60–40	3319	500	3794	402	4252	334
	1481	1900	1006	1998	438	2176
70–30	2993	692	3150	490	3198	300
	607	1108	450	1310	222	1680
10-fold cross validation	964	191	1045	140	1155	68
	236	409	155	460	55	522

**Table 4:** Classification accuracy comparison for stress classification. The table shows maximum (Max), average (avg), and minimum (Min) stress classification accuracy obtained with different methods.

Method	Validation method	Accuracy		
		Max	Avg	Min

MLP	50–50	70.11	69.33	68.56
LSTM 1	50–50	83.33	81.73	80.14
LSTM 2	50–50	87.22	85.79	84.36
MLP	60–40	73.48	71.80	70.12
LSTM 1	60–40	84.69	82.75	80.76
LSTM 2	60–40	89.28	88.22	87.16
MLP	70–30	75.71	73.77	71.84
LSTM 1	70–30	85.82	83.14	81.87
LSTM 2	70–30	90.33	91.11	91.89
MLP	10-fold cross validation	76.27	72.01	70.59
LSTM 1	10-fold cross validation	87.61	86.55	85.97
LSTM 2	10-fold cross validation	93.17	91.96	90.76

**Table 5:** Performance metrics for stress classification using various classification techniques and training-testing set partitions.

Method	Validation method	Specificity	Recall	F1-score	Precision

MLP	50–50	50.17 ± 4.97	79.06 ± 2.35	73.17 ± 3.35	67.66 ± 4.66
LSTM 1	50–50	61.86 ± 3.23	84.67 ± 4.16	79.17 ± 4.73	74.33 ± 4.79
LSTM 2	50–50	88.04 ± 4.21	86.81 ± 4.84	90.04 ± 2.11	93.53 ± 3.23
MLP	60–40	51.19 ± 5.63	82.90 ± 3.43	73.83 ± 4.43	65.14 ± 4.34
LSTM 1	60–40	63.51 ± 3.24	85.41 ± 5.23	80.34 ± 4.39	75.04 ± 5.66
LSTM 2	60–40	86.69 ± 4.44	90.66 ± 4.86	91.68 ± 3.91	82.72 ± 4.27
MLP	70–30	60.60 ± 4.65	79.01 ± 2.28	64.51 ± 3.25	77.26 ± 6.32
LSTM 1	70–30	70.43 ± 4.44	82.16 ± 4.53	83.01 ± 3.93	81.90 ± 6.36
LSTM 2	70–30	84.85 ± 3.72	93.51 ± 3.18	92.45 ± 2.68	91.42 ± 5.23
MLP	10-fold cross validation	61.41 ± 2.23	79.12 ± 3.01	76.81 ± 3.11	76.33 ± 4.69
LSTM 1	10-fold cross validation	71.79 ± 3.65	84.96 ± 4.67	84.62 ± 4.37	82.08 ± 5.53
LSTM 2	10-fold cross validation	88.47 ± 3.42	95.45 ± 2.32	94.94 ± 3.76	94.44 ± 4.43

**Table 6:** Mann-Whitney test based comparison of *p-*values for LSTM 2.

	Training-testing partition	MLP		LSTM 1	
		*p*-value	Significance	*p*-value	Significance
LSTM 2	50–50	2.72 × 10^−11^	Highly significant	1.19 × 10^−9^	Highly significant
	60–40	2.54 × 10^−11^	Highly significant	1.76 × 10^−9^	Highly significant
	70–30	2.37 × 10^−11^	Highly significant	2.25 × 10^−9^	Highly significant
	10-fold cross validation	3.02 × 10^−11^	Highly significant	1.13 × 10^−9^	Highly significant

**Table 7:** Comparison of stress classification accuracies.

Reference	Number of subjects	Number of electrodes	Levels of stress	Stimulus	Classification method	Accuracy (%)

[31]	18	32	2	Emotional video clips	Mean asymmetry scores	—
[37]	9	14	2 and 4	SCWT	SVM	85.17 (2 classes)
						67.06 (4 classes)
[61]	12	3	3	SCWT	SVM	72.3
[35]	7	14	3	Multitasking, SCWT	SVM	77.53
				Arithmetic calculations and memory		
[62]	28	4	2 and 3	Public speaking	MLP	92.85 (2 classes)
						64.28 (3 classes)
[63]	10	1	2	SCWT	SVM	97.6
[21]	9	14	2	High and low altitude	FC-DNN	86.62
				Construction site		
Proposed	35	4	2	Emotional video clips	LSTM 2^1^	87.22
Proposed	35	4	2	Emotional video clips	LSTM 2^2^	89.28
Proposed	35	4	2	Emotional video clips	LSTM 2^3^	90.33
Proposed	35	4	2	Emotional video clips	LSTM 2^4^	93.17

^1^Result for 50–50 training-testing data, ^2^result for 60–40 training-testing data, ^3^result for 70–30 training-testing data, and ^4^result for 10-fold cross validation.

## Data Availability

The data will be made available on request by the corresponding author.
